# From the Operating Theater to the Pathology Laboratory: Failure Mode, Effects, and Criticality Analysis of the Biological Samples Transfer

**DOI:** 10.3390/healthcare12222279

**Published:** 2024-11-14

**Authors:** Francesco De Micco, Anna De Benedictis, Roberto Scendoni, Vittoradolfo Tambone, Gianmarco Di Palma, Rossana Alloni

**Affiliations:** 1 Research Unit of Bioethics and Humanities, Department of Medicine and Surgery, Università Campus Bio-Medico di Roma, 00128 Roma, Italy; f.demicco@policlinicocampus.it (F.D.M.); v.tambone@unicampus.it (V.T.); r.alloni@policlicnicocampus.it (R.A.); 2 Department of Clinical Affair, Fondazione Policlinico Universitario Campus Bio-Medico, 00128 Roma, Italy; a.debenedictis@policlinicocampus.it (A.D.B.); g.dipalma@policlinicocampus.it (G.D.P.); 3 Research Unit of Nursing Science, Department of Medicine and Surgery, Università Campus Bio-Medico di Roma, 00128 Roma, Italy; 4 Department of Law, Institute of Legal Medicine, University of Macerata, 62100 Macerata, Italy; 5 Italian Network for Safety in Healthcare (INSH), Coordination of Marche Region, 62100 Macerata, Italy

**Keywords:** surgical specimens, failure mode, effects and criticality analysis, patient safety, healthcare risk management

## Abstract

*Introduction*: The frozen section intra-operative consultation is a pathology procedure that provides real-time evaluations of tissue samples during surgery, enabling quick and informed decisions. In the pre-analytical phase, errors related to sample collection, transport, and identification are common, and tools like failure mode, effects, and criticality analysis help identify and prevent risks. This study aims to enhance patient safety and diagnostic quality by analyzing risks and optimizing sample management. *Materials and Methods*: The failure mode, effects, and criticality analysis was conducted by a multidisciplinary team to analyze the workflow of frozen section sample handling from collection in the operating theater to acceptance at the pathology lab. Six steps were identified, each assigned tasks and responsibilities, with risks assessed through the risk priority number, calculated from severity, occurrence, and detectability. Severity was classified based on the WHO framework, ranging from “No Harm” to “Death”, to prioritize risks effectively. *Results*: The study identified 12 failure modes across 11 sub-processes, prioritized by risk. Key failures included missing patient identification, incorrect sample retrieval, missing labels, misdirected samples, and samples sent to the wrong lab. *Discussion*: Pre-analytical errors in pathology pose risks to diagnosis and patient care, with most errors occurring in this phase. A multidisciplinary team identified key issues, such as sample mislabeling and delays due to staff unavailability, and implemented corrective actions, including improved signage, staff re-training, and sample tracking systems. Monitoring and regular checks ensured ongoing adherence to protocols and reduced the risks of misidentification, transport delays, and procedural errors. *Conclusions*: The frozen section intra-operative consultation is vital in surgical pathology, with the pre-analytical phase posing significant risks due to potential errors in sample handling and labeling. Failure mode, effects, and criticality analysis has proven effective in identifying and prioritizing these failures, despite resource demands, by allowing corrective actions that enhance patient safety and healthcare quality.

## 1. Introduction

Frozen section intra-operative consultation is a key procedure in the field of pathology allowing real-time evaluation of tissue samples during surgery [1]. This procedure, by providing immediate and crucial information regarding the nature of a pathological tissue, the extent of a lesion, the staging of a cancer, and the appropriateness of excision, helps surgeons make timely and well-informed decisions during surgery [2].

The pathology laboratory (PL) workflow can be divided into three chronologically successive phases: pre-analytical, analytical, and post-analytical [3]. Each of the individual steps can be a source of error [4]; however, the purpose of this study is a risk analysis of only the pre-analytical phase of the frozen section intra-operative consultation.

The pre-analytical step starts with the collection of the sample and ends with the acceptance of the sample at the PL. At this stage, possible errors include misidentification of the specimen, the manner of collection, storage, transport, and acceptance of the sample [3].

PL workflow, involving multiple transfers and passages of samples, has the highest identification error rate [5]. Makary MA et al. showed that identification errors in the operating room occurred in 4.3 cases per 1000 surgical samples. For this reason, health organizations and scientific societies have drawn up strict procedures to be followed for the correct identification of patients and biological samples [6,7].

The collection, storage, and transport of the sample is a further critical point. There are many potentials for error when considering the large number of samples and the numerous steps that a biological sample can take from surgical excision to acceptance at the PL [8]. For these reasons, a cornerstone of the error prevention program for the PL is the traceability of the biological sample, which makes use of systems that enable the automatic recording of sample movements at the various stages and areas of the laboratory [9,10]. The Italian Ministry of Health recommends that hospitals develop and implement procedures for the correct way of transporting biological material from the operating theater to the PL [11]. The College of American Pathologists (CAP) specifies that the policies and procedures of healthcare institutions should outline the timing, method of storage during collection and transport, data, and chain of custody information [7].

Despite the recommendations published by academic and scientific institutions and societies, errors in the identification, collection, and transport of biological samples pose a known safety risk, leading to sometimes serious adverse outcomes for patients and unnecessary costs resulting from both the rework of investigations and corrective actions [12].

Relevant in this scenario is healthcare risk management, the aim of which is to improve the safety of healthcare by identifying and preventing conditions that could put a patient at risk of an adverse event [13,14].

Risk analysis is a key component of risk management, involving a systematic assessment of near misses and high-risk processes where failures could escalate into sentinel events. Among the proactive tools used to anticipate and evaluate potential impacts within critical processes, failure mode, effects, and criticality analysis (FMECA) and hazard vulnerability analysis (HVA) are two of the most widely applied methodologies. These tools help identify vulnerabilities and prioritize risks, facilitating informed decision-making in high-stakes environments [15,16].

FMECA is a systematic method of prospective investigation into the design of a system, process, etc., which assumes that, regardless of the competence or diligence of the workers, in some situations, errors happen unavoidably and may even be very likely to occur. The purpose of the tool is to examine all possibilities in which a fault, error, or malfunction could occur. Then, it is possible to prioritize the ways in which failures, errors, or malfunctions could occur to redesign the system or process for the better and thus bring the greatest benefits [17]. The outcome to be expected from the application of FMECA is the lowering of the risk of defects/failures caused by a lack of or incorrect consideration of the possibility of failures/failures in the design phase [18].

Previous experiences of proactive clinical risk management using FMECA have been published [19,20]. These research studies have the advantage of sharing with the scientific community how clinical risks and their determinants are identified in specific healthcare environments, the causes of active and latent failures, and the interventions applied to prevent risks.

Our research group also intends to participate in the construction of this ‘database’ of good clinical practice for the improvement of patient safety and the implementation of quality healthcare to help improve collective knowledge, promote safety and reliability, and facilitate the sharing of best practices.

The aim of this study is to define the patient safety risks related to the entire frozen section intra-operative consultation procedure and the possible improvement actions to be implemented to ensure that the diagnostic material arrives at its destination on time and under optimal conditions so that it can be analyzed and to guarantee the reliability of the result.

## 2. Materials and Methods

The FMECA was conducted by a multidisciplinary team consisting of clinical risk management experts, pathologists, medical technicians, hospital transport experts, and nursing coordinators.

**Figure 1:**
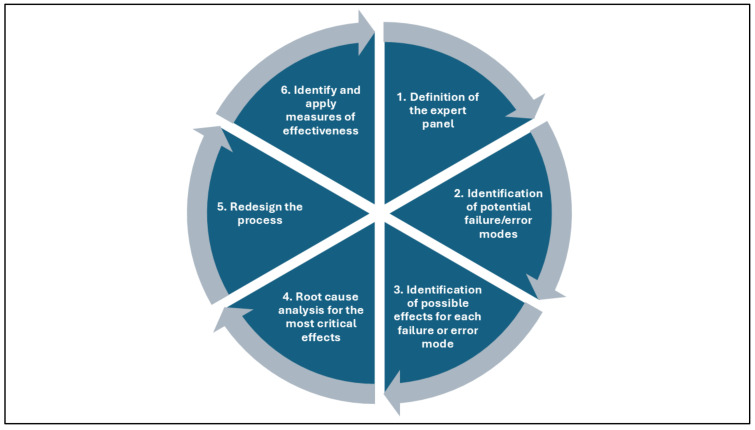
FMECA model: from identifying a high-risk process to redesigning it to reduce risk and ensure greater patient protection.

**Figure 2:**
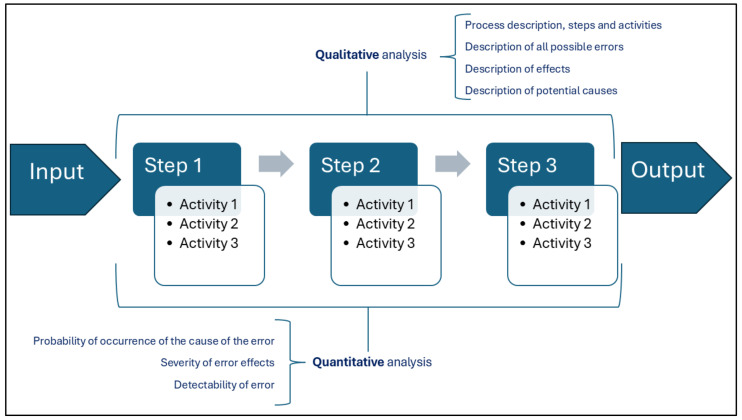
FMECA model: qualitative and quantitative analysis.

According to the FMECA model (Figure 1 and Figure 2) [19], the working group carried out a qualitative and quantitative analysis of the entire workflow, starting with the collection of the surgical sample in the operating theater and ending with the acceptance of the sample at the PL.

**Figure 3:**
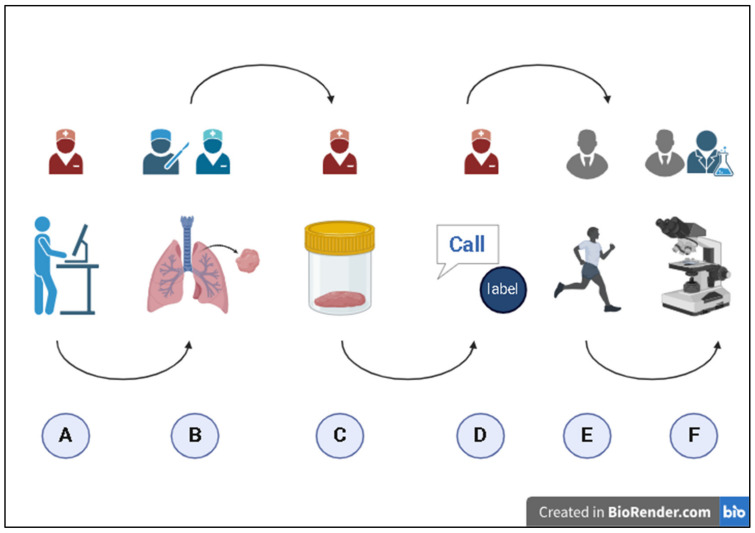
Pre-analytical phase.

The process was divided into six steps identified by a letter (Figure 3):

A. Computer compilation and registration;
B. Collection;
C. Biological sample packaging;
D. Biological sample sent to the PL;
E. Biological sample transport;
F. Biological sample delivery to the PL.

Each stage was subdivided into activities identified by an alphanumeric code, and the healthcare worker responsible was identified for each activity, as shown in Table 1.

For each task, the risk priority number (RPN) was calculated as a product of three characteristics: S, the severity of the effects of the error; O, the probability of the cause of the error occurring, choosing values with reference to the literature [20,21]; and D, the detectability of the error (Table 2). It can take a maximum value of 1000 (10 × 10 × 10: product of the maximum scores) and a minimum value of 1 (1 × 1 × 1: product of the minimum scores). For the assessment of severity of harm, the working group used the World Health Organization’s Conceptual Framework for the International Classification for Patient Safety: (i) No harm: The patient experiences no symptoms, or symptoms are undetectable, with no treatment necessary. (ii) Mild harm: The patient has mild, short-term symptoms or a slight functional impact that requires minimal or no intervention, such as additional observation, review, or minor treatment. (iii) Moderate harm: The patient experiences noticeable symptoms requiring intervention (e.g., additional surgery, therapeutic treatment) or a prolonged hospital stay or results in lasting or permanent harm or loss of function. (iv) Severe harm: The patient’s symptoms necessitate life-saving or major medical intervention, potentially shortening life expectancy or resulting in significant long-term harm or major functional loss. (v) Death: The incident likely led to or expedited the patient’s death in the short term [22].

## 3. Results

### 3.1. Quantitative Process Analysis

The study identified 12 failure modes within the 11 identified sub-processes, as shown in Table 3. Several potential error modes that could compromise the integrity and traceability of pathology specimens were identified during the process analysis. Among these, diagnostic query errors on the HIS system, printing of labels with incorrect patient data, and incorrect label placement (e.g., on the lid instead of the container) were sources of risk to proper identification. Other risks included damage or inadequacy of primary and secondary containers for pathological specimens and the possibility of the specimen being withdrawn from the HIT without the correct identification data, resulting in the collection of a specimen other than the one requested. In addition, the temporary absence of the FSHT can cause delays, while failure to apply the label can result in the sample being mistakenly sent to the medical laboratory instead of the pathology laboratory. These errors, if not managed, can lead to diagnostic delays, patient safety risks, and the need to revise or correct the process. Misassignment of the diagnostic request and report to a different patient can lead to misdiagnosis and treatment, posing serious health and safety risks to the patient involved. In addition, the mismatch between the biological specimen and patient can lead to diagnostic delays and the need to repeat the procedure, increasing the risk of clinical errors. Frequent delays in transport and diagnostic response result in longer anesthesia and surgery times, increasing operative risks to the patient and the possibility of postoperative complications. Timeliness and accuracy in specimen management are therefore essential to minimize these negative implications and ensure the safety and efficacy of the diagnostic–therapeutic pathway.

**Figure 4:**
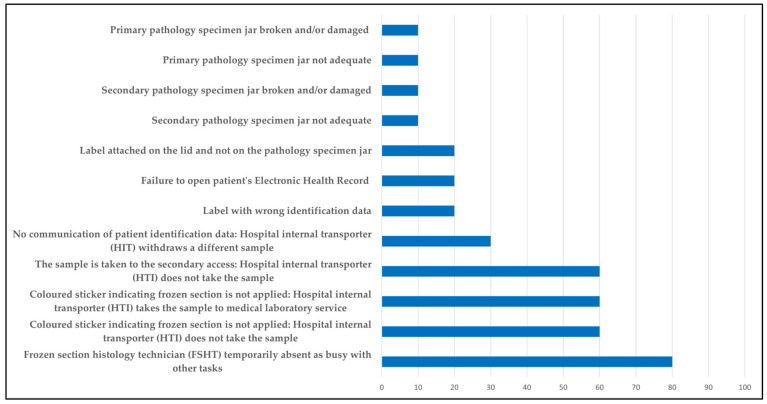
Identified risk priorities.

Each failure mode was ordered by increasing risk priority, as shown in Figure 4.

### 3.2. Patient Safety Improvement Actions

Several improvement actions were implemented to optimize specimen handling and security in the Pathology Department. An information sign was placed at the entrance of the department to provide the name and telephone number of the FSHT on duty, making the necessary information visible to facilitate direct and timely contact. In addition, all HITs were instructed regarding the various rooms in the laboratory where the FSHT could be found in case of absence from the main laboratory dedicated to frozen sections. A dedicated specimen tracking log was introduced, in which the following were recorded for each specimen: patient identification label, specimen number, time, and signature of the CN and HIT for each step. To ensure accuracy in handling, periodic re-trainings were provided for FSHTs and HITs, focusing on sample tracking, collection, transport, and storage procedures. Finally, a double nurse check was instituted before sending samples to the laboratory to minimize the risk of identification errors and ensure proper management of the process. Periodic checks were implemented to ensure proper procedure application and reduce risks of errors in handling histological samples. The monitoring included regular verification of the correct completion of the adopted forms, actively involving the FSHT and HIT to ensure compliance. Additionally, periodic checks of the secondary access designated for pathology specimens were carried out to confirm that containers were properly labeled and tracked. Furthermore, the ratio of inadequate or damaged primary or secondary specimen jars to the total number of jars used was regularly monitored to promptly identify any issues and implement corrective actions, thereby maintaining high standards of safety and quality in sample storage and transport [Table 4].

## 4. Discussion

Pre-analytical errors in pathology are a significant challenge to the correct diagnosis and prognosis of patients [4]. This phase, which precedes the analysis of the biological sample, is characterized by a series of crucial steps, from the request for the examination to the delivery of the sample to the pathologist [3]. The majority of laboratory errors (61.9%) occur in the pre-analytical phase, with 15% happening in the analytical phase and 23.1% in the post-analytical phase [23,24].

Errors at this stage, such as inadequate fixation and incorrect labeling, can compromise the entire procedure and lead to incorrect or delayed diagnoses, with consequent clinical repercussions for the patient [6,7]. Preventing and reducing these errors require constant attention to the standardization of procedures, staff training, and the implementation of effective quality control systems [25]. For these reasons, one of the pillars of the error prevention plan is the traceability of the biological sample, supported by systems that record the movements of the sample at different stages and areas of the laboratory [26]. This creates a detailed trace and reduces the risk of errors related to sample handling [8].

The quantitative analysis of the process showed that one of the most critical points of the process is the possibility that the frozen section histology technician (FSHT) is temporarily absent, busy with other tasks. This could lead to a delay in response and a consequent extension of anesthesia and surgery times. To lower the risk level, an information sign was placed at the PL entrance, exclusively dedicated to frozen section intra-operative consultation, with the name and telephone number of the FSHT on duty for each day of the month. All hospital internal transporters (HITs) were informed of the other rooms in the laboratory where they could find the FSHT if the FSHT was not present in the frozen section laboratory. The improvement action was monitored by periodically checking the correct filling-in of the adopted forms and the involvement of the FSHTs and the HITs. Three sub-processes showed the same level of criticality and could lead to a delay in the transport of the specimen in the response to the surgical question with a consequent extension of anesthesia and surgery time: a colored sticker indicating the frozen section is not applied, and the HIT does not take the sample; a colored sticker indicating the frozen section is not applied, and the HIT takes the sample to the medical laboratory service; and the sample is taken to the secondary access, and the HIT does not take the sample. The assessment made by the working group, because of the qualitative and quantitative analysis, is reflected in the literature. Mislabeling on specimens can lead to patient injuries due to misdiagnosis and delays in diagnosis. A CAP study on errors at the pathology laboratory showed that 19.8% involved specimens, and in 20.9% of cases, the error occurred before acceptance [27]. As an improvement action, the working group proposed a periodic (1 or 2 times/year) re-training for operating room nurses and HITs, with the topics of the procedure being the traceability, collection, transport, and storage of histopathological specimens. Improvement action was also monitored through the periodic verification of the correct filling-in of the adopted forms and periodic inspection of the secondary access to check for pathology specimen jars.

Let us turn to possible errors in specimen identification and handling [28,29]. Errors in the identification of surgical specimens are common and represent significant risks for all patients [30]. Out of a total of 1,004,115 reviewed cases, errors related to sample identification accounted for 9.6 percent, while 3.6 percent concerned sample handling [31]. Therefore, a further sub-process that raised concern in the working group was the possibility of no communication of patient identification data, whereby the HIT withdraws a different sample. The occurrence of this failure would cause a delay in the transport and response to the surgical question, thus prolonging the anesthesia time and the surgery itself. To minimize the risk of failure, the working group introduced a register on which it is mandated for each patient: (a) to affix the patient identification label; (b) to write down the number of samples and the time the sample(s) were deposited and to affix the signature of the circulating nurse (CN) who deposited the sample(s) at the designated place; and (c) to write down the number of samples and the time the sample(s) were collected and to affix the signature of the HIT who collected the sample(s) at the designated place. Improvement action was monitored through regular verification of the correct filling-in of the adopted forms.

The analysis of the remaining sub-processes showed very low risks of error. To further decrease the residual risks of error of a label with wrong identification data, a label attached on the lid and not on the pathology specimen jar, and failure to open the patient’s electronic health record (EHR), the operating room nurses participated in a re-training concerning the traceability, collection, transport, and storage of samples, and the improvement action was monitored by periodically checking the correct filling-in of the adopted forms. Although potentially residual, the working group considered it appropriate to deal with it, as in the event of realization, the request and report would either be attributed to another patient, or a failure to match the biological sample to the patient would be possible.

Finally, a delay in response and a consequent lengthening of the anesthesia and surgery time could be causally related to a primary or secondary pathology specimen jar being not adequate/broken/damaged, which was addressed by introducing an experimental double-checking by nurses before sending the samples to the laboratory. The improvement was monitored by periodically checking the ratio of the primary or secondary pathology specimen jars that were not adequate/broken/damaged to the number of primary or secondary pathology specimen jars used. Overall, the improvement actions and related monitoring activities, schematized in Table 4, aimed to implement the process by which it is possible to ensure that a biological sample is correctly identified, managed, and documented during all stages of the process.

## 5. Conclusions

Frozen section intra-operative consultation is an essential step in surgical pathology, allowing immediate analysis of tissue samples during surgery. The pre-analytical phase that starts with the creation of the request on the HIS, the printing of the labels with the patient’s data, and the labeling of the container and ends with the delivery of the biological sample to the PL can present significant issues. Given the frequency and potential impact of errors at this stage, tracking the error rate is a key indicator of patient safety. Reducing these errors should be a priority in research, as it is essential in many other scenarios involving sample transfer and analysis [32].

Also in this field, FMECA has proved to be a valuable tool for identifying and evaluating potential failures in a process and for determining their consequences and criticality. The use of systematic methodology enabled the working group to identify how each component of the overall process could fail, to analyze the consequences for patient safety, and to assess the severity of the effects. Even with the limitations widely known in literature, such as the need for an investment in time and resources, and the close correlation between team member experience and improvement actions, FMECA enabled us to identify certain weaknesses in the pre-analysis phase, prioritize and formulate corrective and preventive actions, and contribute to improving the quality and safety of healthcare.

## Figures and Tables

**Table 1:** Qualitative process analysis.

Phase		Activity	Responsibility
**A.** Computer compilation and registration	A1	Diagnostic examination request via HIS	CN
	A2	Printing of labels with patient data	CN
	A3	Container labeling	CN
**B.** Collection	B1	The biological sample is delivered from the surgeon to the IN	Surgeon; IN
	B2	The biological sample is delivered from the IN to the CN	IN; CN
**C.** Biological sample packaging	C1	The biological sample is collected in the primary pathology specimen jar	CN
	C2	The biological sample is collected in the secondary pathology specimen jar	CN
**D.** Biological sample sent to the laboratory	D1	The CN contacts the Hospital Internal Transporter Service notifying the collection of a biological sample for frozen section intra-operative consultation	CN
	D2	The label identifying biological samples for frozen section intra-operative consultation is attached by the CN	CN
	D3	The biological sample is taken by the CN outside the operating room and stored in a room designated for this purpose	CN
**E.** Biological sample transport	E1	The HIT picks up and transports the sample to the PL	HIT
**F.** Biological sample delivery to the PL	F1	The HIT hands over the biological sample to the FSHT	HIT

HIS: Hospital information system; IN: instrumental nurse; FSHT: frozen section histology technician; HIT: hospital internal transporter; CN: circulating nurse; PL: pathology laboratory. Each stage was subdivided into activities identified by an alphanumeric code (A1, A2, A3, …, F1).

**Table 2:** Severity of the event (S), frequency of the event occurring (O), detection of the error (D), and relative score system.

Severity (S)	Occurrence (O)	Detection (D)	Score
No harm Mild harm Moderate harm	Very low (1:10,000)	Very high (9:10)	1–2
	Low (1:5000)	High (7:10)	3–4
	Moderate (1:200)	Moderate (5:10)	5–6
Severe harm	High (1:100)	Low (2:10)	7–8
Death	Very high (1:20)	Very low (<1:10)	9–10

**Table 3:** Quantitative process analysis.

Activity	Failure Mode	Implications for Patient Safety	S	O	D	RPN
A1	Incorrect diagnostic examination request via HIS	Request and report assigned to the wrong patient	5	2	2	20
A2	Printing of labels with wrong patient data	Request and report assigned to the wrong patient	5	2	2	20
A3	Label placed on the lid and not on the container jar	Failure to match biological sample to patient	10	1	2	20
B1	No risk of failure	Not applicable	0	0	0	0
B2	No risk of failure	Not applicable	0	0	0	0
C1	Primary pathology specimen jar broken and/or damaged and/or not adequate	Delay in transport and response, resulting in longer time for anesthesia and surgery	10	1	1	10
C2	Secondary pathology specimen jar broken and/or damaged and/or not adequate	Delay in transport and response, resulting in longer time for anesthesia and surgery	10	1	1	10
D1	Failure to provide patient identification data. HIT withdraws a different sample from the one requested	Delay in transport and response, resulting in longer time for anesthesia and surgery	10	3	1	30
D2	The label is not applied	Delay in transport and response, resulting in longer time for anesthesia and surgery	10	3	2	60
D3	The biological sample is taken to the secondary access. HIT does not take the sample	Delay in transport and response, resulting in longer time for anesthesia and surgery	10	3	2	60
E1	HIT takes the biological sample to the medical laboratory instead of PL due to lack of label	Delay in transport and response, resulting in longer time for anesthesia and surgery	10	3	2	60
F1	FSHT temporally abs	Delay in transport and response, resulting in longer time for anesthesia and surgery	10	4	2	80

HIS: Hospital information system; HIT: hospital internal transporter; PL: pathology laboratory; FSHT: frozen section histology technician. Each error mode is identified by an alphanumeric code corresponding to the activity identified in the qualitative analysis (A1, A2, A3, …, F1).

**Table 4:** Improvement and monitoring actions.

Actions	Improvement Actions	Monitoring Actions
F1	At the PL entrance, a dedicated frozen section information sign was placed with the name and telephone number of the FSHT on duty for each day of the month. All HITs were informed of the other rooms in the laboratory where they could find the FSHT if they were not present in the laboratory dedicated to the frozen sections.	Periodic check of the correct filling-in of the adopted forms and involvement of the FSHTs and HITs.
D2	Periodic re-training for operating room nurses and HITs (1–2 times/year) regarding the procedure for the traceability, collection, transport, and storage of histopathological samples.	Periodic check of the correct filling-in of the adopted forms.
D3	Periodic re-training for operating room nurses and HITs (1–2 times/year) regarding the procedure for the traceability, collection, transport, and storage of histopathological samples.	Periodic check of the proper application of the procedure. Periodic check of the secondary access for pathology specimen jars.
E1	Periodic re-training for FSHTs and HITs (1–2 times/year) regarding the procedure for the traceability, collection, transport, and storage of histopathological samples.	Periodic check of the correct filling-in of the adopted forms.
D1	Introduction of a register on which for each patient it is mandated: (a) to affix the patient identification label; (b) to write down the number of samples, the time the sample(s) was deposited, and to affix the signature of the CN who deposited the sample(s) at the designated place; (c) to write down the number of samples, the time the sample(s) was collected, and to affix the signature of the HIT who collected the sample(s) at the designated place.	Periodic check of the correct filling-in of the adopted forms.
A1	Periodic re-training regarding the procedure for the traceability, collection, transport, and storage of histopathological samples.	Periodic check of the correct filling-in of the adopted forms.
A2	Periodic re-training regarding the procedure for the traceability, collection, transport, and storage of histopathological samples.	Periodic check of the correct filling-in of the adopted forms.
A3	Periodic re-training regarding the procedure for the traceability, collection, transport, and storage of histopathological samples.	Periodic check of the correct filling-in of the adopted forms.
C1	Double nursing check before sending samples to the PL.	Periodically checking the ratio of the primary or secondary pathology specimen jar not adequate/broken/damaged to the number of primary or secondary pathology specimen jars used.
C2	Double nursing check before sending samples to the PL.	Periodically checking the ratio of the primary or secondary pathology specimen jar not adequate/broken/damaged to the number of primary or secondary pathology specimen jars used.

FSHT: Frozen section histology technician; HIT: hospital internal transporter; CN: circulating nurse; PL: pathology laboratory. Each improvement and monitoring action is identified by an alphanumeric code corresponding to that used in the qualitative and quantitative analysis (A1, A2, A3, …, F1).

## Data Availability

No new data were created or analyzed in this study. Data sharing is not applicable to this article.
